# 25-vitamin D reduces inflammation in uremic environment

**DOI:** 10.1038/s41598-019-56874-1

**Published:** 2020-01-10

**Authors:** Rodrigo Barbosa de Oliveira Brito, Jacqueline Ferritto Rebello, Caren Cristina Grabulosa, Walter Pinto, Armando Morales, Rosilene Motta Elias, Rosa Maria Affonso Moyses, Maria Aparecida Dalboni

**Affiliations:** 1 Universidade Nove de Julho, UNINOVE, Sao Paulo, Brazil; 2 Hospital das Clinicas HCFMUSP, Universidade de Sao Paulo, Sao Paulo, Brazil

**Keywords:** Applied immunology, Haemodialysis

## Abstract

Chronic kidney disease (CKD) is characterized by loss of renal function and a consequent increase of serum uremic toxins, which contribute to inflammation status. Deficiency of 25-vitamin D, often found in patients with CKD, has been included as an inflammatory factor since it might modulate the immune system. The aim of this study was to investigate the role of 25-vitamin D on inflammatory pathways in healthy and uremic environment. Toll-like receptor 4 (TLR4), oxidative stress (ROS), vitamin D receptor (VDR), 1-α hydroxylase (CYP27), 24 hydroxylase, cathelicidin, and MCP-1 were evaluated in monocytes exposed to a uremic serum pool compared with healthy pool. The human monocytes lineage (U937) was incubated with or without 25-vitamin D (50 ng/ml for 24 hours). TRL4, VDR, CYP27, CYP24, and ROS were evaluated by flow cytometry. We used ELISA to measure IL-6, TNF-α, IL-10, cathelicidin, and MCP-1 in the cell culture supernatant. We observed a higher expression of TRL-4, IL-6, TNF-α, IL-10, cathelicidin and MCP-1 in monocytes incubated with uremic serum when compared with serum from healthy individuals. Supplementation of 25-vitamin D was able to reduce the expression of TRL4, cathelicidin, and MCP-1 in the uremic environment. There was no difference in the expression of VDR, CYP27 and CYP24 intracellular enzymes. This *in vitro* study showed that the uremic pool activates inflammatory response in monocytes, which was reversed by 25-vitamin D supplementation; this finding suggests that 25-vitamin D has an anti-inflammatory role in the uremic environment.

## Introduction

Chronic Kidney disease (CKD) is characterized by the loss of glomerular filtration rate resulting in serum accumulation of toxic substances, called uremic toxins^1^. The accumulation of these compounds is defined as the uremic environment.

It has been shown that uremic toxins are capable to induce an inflammatory response in patients with CKD^2–4^. Uremic toxins can cause oxidative stress and stimulate the production of proinflammatory cytokines in this population. Several studies have been reported a relationship between uremic toxins (mainly indoxyl sulfate, p-cresyl sulfate, and indole-3-acetic acid) and inflammation^3,5,6^. The indoxyl sulfate has been described to activate NF-ĸB, NADPH oxidase, upregulated mRNA and expression of intercellular adhesion molecule-1 (ICAM-1)^7–9^, and is also capable to induce endothelial injury with the formation of microvesicles that contribute to endothelial cell progenitors dysfunction^10^. The p-cresyl sulfate and indole-3-acetic acid are associated with increased IL-6 and CRP, respectively, in patients with CKD^3,11^.

When renal function deteriorates to <15 ml/min/1,73 m^2^, patients usually need dialysis support. However, despite the technological advances in the dialysis procedures, thrice weekly conventional hemodialysis is not able to remove all toxins, particularly those too large and/or protein-bounded^12^. Therefore, patients on dialysis still have serum circulating uremic toxins. Some studies have demonstrated a direct relationship between uremic toxins and inflammation and cardiovascular disease, which is the main cause of mortality in patients with CKD^13,14^.

Uremic toxins can cause immune activation with production of proinflammatory cytokines including TNF-α, IL-6 and MCP-1^15,16^ mainly by monocytes^17,18^. Although these proinflammatory cytokines enhance host defense, their excessive production leads to unresolved inflammation^19^. The uremic toxins may induce the toll-like receptor (TLR) activation, resulting in the production of several inflammatory mediators^20^. Our group has recently reported that TLR4 expression is increased in neutrophils and monocytes obtained from hemodialysis patients that correlated with IL-6, reinforcing the role of TLR4 in the mechanism of inflammation^21^.

Besides cytokines, 25-vitamin D has also been implicated as an inflammatory marker, participating in both innate and adaptive immunity^22^. Hypovitaminosis D has been often reported in patients with CKD, reaching up to 80% of prevalence^23^. Although the levels of 1,25-vitamin D are important, it is the circulating concentration of 25-vitamin D that determines the vitamin D status of a given individual. Levels of serum 25-vitamin D from 20 to 60 ng/mL are considered normal and values below 20 ng/mL are considered indicative of vitamin D deficiency^24^.

The vitamin-D receptor (VDR) and the enzyme 1α-hydroxylase (CYP27) are present in cells of the immune system^25–27^. 25-vitamin D regulates the expression of cathelicidin, an endogenous antimicrobial peptide^28^. This modulation occurs by activation of TLRs, which sign for increased expression of VDR and CYP27, the enzyme that converts 25-vitamin D to the active form, 1,25-vitamin D. This active form regulates the VDR that sign the hCAP18 encoding gene, which is a pro-protein that upon be cleaved releases cathelicidin^29^ to act against gram-negative and positive bacteria, viruses and fungi^30^.

Uremic toxins and hypovitaminosis D are important inflammatory markers in patients with CKD. However, few studies evaluated the effect of supplementation of 25-vitamin D on the cells and the mechanisms involved in the innate immunity response. Therefore, the goal of the current study was to evaluate the *in vitro* effect of 25-vitamin D supplementation on inflammation through monocytes cells. The expression of the receptors TLR4, VDR, 1-α hydroxylase, cathelicidin, in addition to oxidative stress and inflammatory cytokines were also evaluated.

## Matherial and Methods

Culture assay: The U937 lineage^31^ (human monocytic lineage, ATCC #TIB-202) was differentiated into monocytes using phorbol 12-myristate 13- acetate (PMA; Sigma-Aldrich P1585) to evaluate the modulation of 25-vitamin D on the expression of TLR4, VDR, CYP27, CYP24, cathelicidin, IL-6, IL-10, MCP-1, and oxidative stress, as previously described^32^. U937 was cultured in RPMI 1640 with (pH 7.4, Sigma Chemical Co., St. Louis, MO, USA), 10 mmol/L HEPES (Sigma Chemical Co., St. Louis, MO, NY, USA), 2 mmol/L L-Glutamine (Merck, Darmstadt, Germany), 100 U/mL penicillin and 100 μg/mL streptomycin (Gibco, BRL, Life Technologies, USA), 10% fetal bovine serum and maintained in culture with 5% CO_2_ incubator at 37 °C.

The culture medium was changed every 48 hours until the cell concentration reached 1 × 10^6^/mL. Subsequently, the cells were stimulated by 10 nM/mL PMA for 24 hours in a 75 cm^3^ culture bottle containing 1 × 10^6^ cells/mL. After differentiation, the cells were washed in 10 mL of PBS (Sigma, Cat P3744-1PAK) to remove the PMA and incubated with 5 mL of 0.5% trypsin, in order to detach the cells from the surface of the plaque, obtaining a homogeneous cell suspension. The cells were then washed with culture medium (5 mL) for trypsin neutralization, and cell viability testing was performed using Trypan Blue.

After differentiation, U937 cells were transferred to culture bottles of 25 cm^2^, preincubated with 25-vitamin D (Sigma-Aldrich CAT: 101443236) at the concentration of 50 ng/mL, for 24 hs. Afterward, the cells were incubated with serum from uremic patients or serum from healthy subjects. Detailed description of Monoclonal antibodies is shown in Supplementary Table 1.

### Preparation of human serum pool

Healthy and uremic serum pool were prepared according to our previous study^33^, as follows:

*Healthy Serum Pool*: Blood samples (20 mL) were collected from 20 healthy volunteers in tubes with anticoagulants and centrifuged at 500 G for 10 minutes. The supernatant was stored in freezer aliquots −80 °C.

*Uremic Serum Pool:* blood samples were collected with anticoagulant tubes from 30 patients in hemodialysis treatment imediately before the begining of the second session of the week. The tubes were centrifuged at 500 G for 10 minutes. The supernatant was stored in a freezer −80 °C. Patients that had any type of infection, use of immunosuppressive drugs, vitamin D supplementation, diabetes mellitus or neoplasia disease were excluded from the study.

An aliquot from both sample pools were sent to the laboratory to be sure that uremic pool was representative of uremic environment, detected by the increase of levels of creatinine, urea, parathyroid hormone (PTH), phosphorus and decrease of calcium and 25-vitamin D. Besides, we performed the Limulus amebocyte lysate test (LAL) on both uremic and healthy pool serum to evaluated lipopolysaccharide (LPS) levels, which cause monocyte activation in levels >0.25mlU/mL.

This study was approved by the Institutional Review Board of the Federal University of São Paulo (#0727/10). Healthy and uremic serum samples were obtained after written informed consent of participants or legal representative, according to the Helsinki Declaration and local regulations.

### Expression of CD14, TLR4, VDR, CYP27 and CYP24 by flow cytometry

3 × 10^5^ cells from healthy and uremic serum (pre-incubated or not with 25 vitamin D) were used to label monoclonal antibodies to CD14-FITC and TLR4 PE and VDR-APC, CYP27-Alexa Fluor 647 and CYP24-Alexa Fluor 488 (Supplementary Table 2) according to manufactures instructions, and was added to healthy and uremic serum pool.

The expression of these monoclonal antibodies was checked by the flow cytometer (FacsCanto I, BD, USA) immediately.

### Flow cytometry analysis

A forward scatter plot versus side scatter plot was used to make a gate for the U937 cells and to exclude debris. DOT-PLOT graphs were used to analyze the CD14, TLR-4 and VDR (Supplementary Fig. 1) and histograms were used to analyze the CYP27 and CYP24 (Supplementary Fig. 2) and ROS (Supplementary Fig. 3. The results were described in mean fluorescence intensity (MFI), such that the higher the MFI the greater the expression of these receptors.

### Detection of reactive oxygen species (ROS) by Flow Cytometry

The 3 × 10^5^ cells each condition were transferred into the 12 × 75 mm tube and incubated with 100 uL of 2′-7′-dichlorofluorescein diacetate (DCFH-DA)(FITC) (Sigma, St. Louis, MO, USA) to evaluated ROS, according to previously described^34^. The analyses were performed by the flow cytometer (FacsCanto I, BD).

### Detection of TNF-α, IL-6, IL-10, cathelicidin, MCP-1 by ELISA technique

The culture supernatants were stored at −80 °C freezer to detection TNF-α, IL-6, IL-10, cathelicidin and MCP-1 by enzyme-linked immunosorbent assay (ELISA) assays. ELISA tests were performed using commercial Kits according to manufacturers’ instructions (Supplementary Table 1).

### Detection of NF-κB

The monocytes were evaluated for NF-κB activity, according to the manufacturer’s instructions (NF-κB p50/p65 Transcription Factor Assay Kit, eBioscience, San Diego, USA). The respective inter- and intra-assay coefficient of variation was 4.1% and 5.9%.

### Statistical analysis

The results according to each group (healthy, uremic, with 25-vitamin D supplementation and without 25-vitamin D supplementation) were expressed as median (minimum and maximum), and the differences tested by Kruskal Wallis. Relationships between independent variables were performed in the entire group. Kolmogorov-Smirnov test was used to test normality of data. We used Pearson or Spearman correlation coefficient, as appropriate. The value of p < 0.05 was considered for statistical significance. The analyses were performed using SPSS (Statistical Package Social Sciences) software version 22.0 for Windows.

## Results

Comparison of characteristics between healthy and uremic pool:

Biochemical characteristics: As expected, uremic pool had higher creatinine, urea, PTH, and phosphorus, whereas serum calcium and 25-vitamin D were lower (Table 1).

**Table 1:** Biochemical data from healthy and uremic serum pool.

	Healthy Pool	Uremic Pool
Creatinine (mg/dL)	0.82	8.92
Urea (mg/dL)	33	135
PTH (pg/mL)	23	392
Calcium (mg/dL)	9.7	7.4
Phosphorus (mg/dL)	3.4	4.3
25-vitamin D (ng/mL)	19.2	10.5
LPS levels (mlU/mL)	<0.25	<0.25

PTH, parathyroid hormone; LPS, lipopolisaccharide.

Inflammatory markers: We observed a higher expression of TRL4, ROS, IL-6, TNF-α, IL-10, cathelicidin, and MCP-1 in monocytes incubated with uremic serum when compared to healthy serum (Table 2). When monocytes were incubated with uremic serum previously treated with 25-vitamin D, there was a reduction in the expression of TLR4, MCP-1, and cathelicidin. There were no differences regarding the expression of VDR, CYP27, CYP24, and NF-κB between groups (Table 2).

**Table 2:** Expression of TLR-4, ROS, VDR, CYP27, CYP24, IL-6, TNF-α, IL-10, cathelicidin, MCP-1 and NF- κB.

	Healthy pool (HS)		Uremic pool (US)	
	HS	HS + 25 vitamin D	US	US + 25 vitamin D
**Expression (MIF)**				
TLR4	716 (654–936)	644 (644–870)	2653* (1853–3372)	1692^†^ (1022–2292)
ROS	343 (313–498)	378 (320–563)	550* (394–607)	544 (435–599)
VDR	7882 (6574–9687)	8394 (6475–10708)	7657 (6670–9420)	7556 (5699–9420)
CYP27	1201 (692–1461)	1207 (747–1521)	1396 (931–1532)	1170 (955–1521)
CYP24	439 (319–563)	363 (329–501)	342 (331–676)	375 (333–485)
**Cytokines (pg/mL)**				
IL-6	3.8 (3.3–4.9)	3.6 (3.1–5.3)	39.3* (28.3–45.3)	37.3 (28.8–42.5)
TNF-α	2.2 (1.6–2.7)	2.3 (2.1–3.2)	3.9* (3.2–4.7)	3.4 (2.9–4.8)
IL-10	45 (41–62)	47 (45–62)	61* (53–64)	60 (56–73)
CATHELICIDIN	0.44 (0.43–0.46)	0.43 (0.4–0.49)	0.58* (0.51–0.65)	0.52^†^ (0.48–0.59)
MCP-1	11076 (7899–11948)	7336 (4820–8905)	14370* (9117–16936)	7697^†^ (5940–10310)
NF- κB (%)	4.8 (1.6–1.2)	3.1 (1.7–3.4)	3.4 (2.1–5.5)	4.1 (2.3–6.4)

*US ≠ HS: p < 0.04.

^†^US + 25 vitamin D ≠ US: p < 0.03.

**Figure 1:**
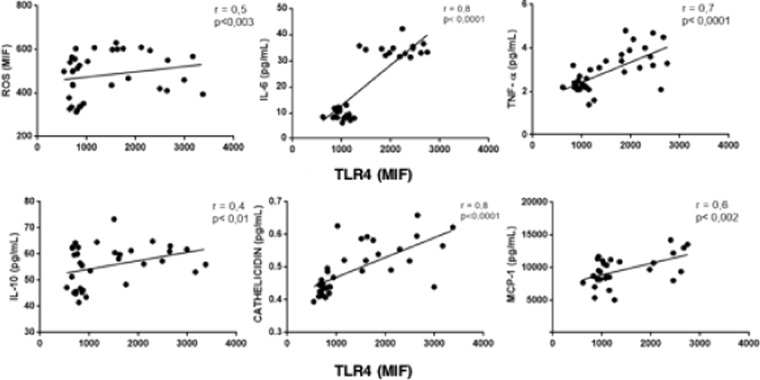
Correlation between TLR-4 and ROS, IL-6, TNF-α, IL-10, cathelicidin and MCP-1.

**Figure 2:**
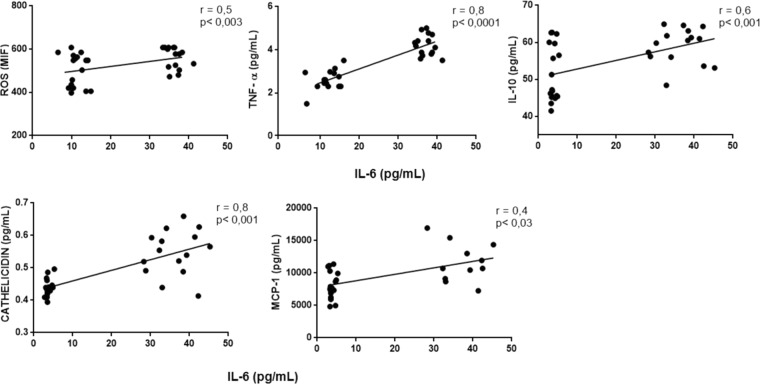
Correlation between expression of IL-6 and ROS, TNF-α, IL-10, Cathelicidin and MCP-1.

**Figure 3:**
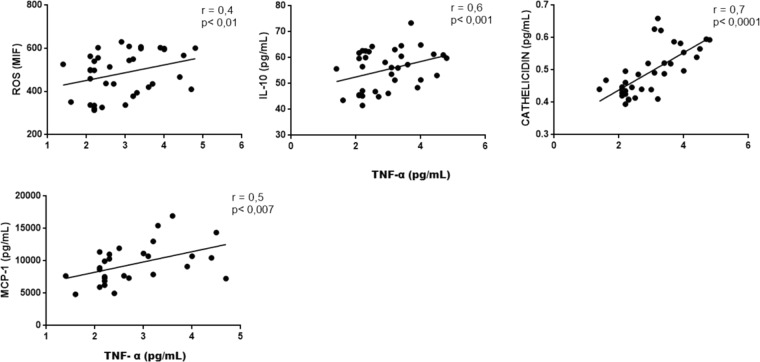
Correlation between expression of TNF-α and ROS, IL-10, cathelicidin and MCP-1.

**Figure 4:**
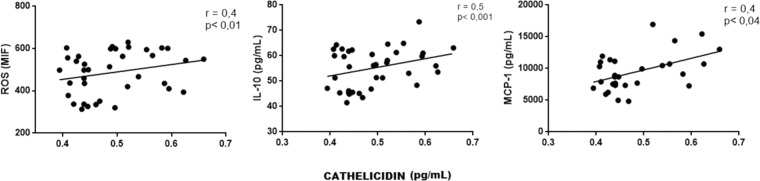
Correlation between expression of Cathelicidin and ROS, IL-10 and MCP-1.

Correlations among inflammatory markers: We performed correlation between TLR4 and other variables to address the interplay among this receptor and inflammatory markers. In fact, there was a positive correlation between the expression of TLR4 and ROS, IL-6, TNF-α and IL-10, MCP-1 and cathelicidin (all p values < 0.05). There was also a positive correlation between IL-6 with ROS, IL-10, cathelicidin and MCP-1, a positive correlation between TNF-α and ROS, IL-10, cathelicidin and MCP-1, and between cathelicidin and ROS, IL-10 and MCP-1 (all p values < 0.05). These correlations are illustrated in Figs. 1, 2, 3 and 4).

## Discussion

In this study, we confirmed that the uremic environment is associated with an inflammatory status, which was depicted by elevated levels of cytokines and ROS. In addition, uremic serum, similarly to what happens in patients with CKD presented low levels of 25-vitamin D. The novelty of our study, however, was demonstrating that 25-vitamin D was capable to reduce TLR-4, MCP-1, and cathelicidin in a uremic environment. Taken together, our findings provide evidence that vitamin D has a role in protecting against inflammation, at least mediated by monocytes.

The high TLR4 expression is important to improve the infection response, however the continuous expression leads to a release of pro-inflammatory cytokines. We observed a high expression of TLR4 in monocytes incubated with uremic serum compared with healthy serum and a positive association with cytokines and ROS. The TLR4 expression may activate the intracellular nuclear factor-κB (NF-κB) pathway and enhances the expression of NF-κB-controlled genes such as for inflammatory cytokines and adhesion molecules not only in monocytes but also in endothelial cells^35,36^. In our *in vitro* study, we did not observe a difference in the percentage of NF-κB, despite the overexpression of TLR4 in monocytes incubated with uremic serum.

The short-time incubation could lead to this contradictory result, although our results are similar to a previous study that did not show an effect of a 16-week supplementation of vitamin D on NF-κB activity^37^. TLR4, however, associated with cytokines, confirming previous studies^21,38^ in patients on hemodialysis.

We observed an increased production and secretion of ROS and MCP-1 in monocytes incubated with uremic serum compared to healthy serum. The Monocyte chemoattractant peptide protein 1 (MCP-1) has been described to attract circulating cells from blood vessels to the local injury sites and together with inflammatory mediators and ROS may contribute to the increased vascular injury^39^ and also cardiovascular disease in patients with CKD^40^. The relationship between uremia and the vascular lesion has been demonstrated by Stinghen A *et al*.^41^, describing in an *in vitro* study that indoxyl sulfate, a uremic toxin, causes endothelial lesion that may contribute to cardiovascular disease in patients with CKD.

The supplementation of 25-vitamin D, tested in the current study as an anti-inflammatory agent, seems to have an effect on monocytes. The lower TLR4 expression suggests a downregulation effect of 25-vitamin D on this receptor. Recently, Zhang Y *et al*.^42^ described a beneficial effect of 25-vitamin D supplementation on the decrease of IL-6 and TNF-α, though this study was conducted in patients without renal disease. We found no effect of the 25-vitamin D supplementation downregulating cytokines that might be related to the short period of treatment (24-hour).

The 25-vitamin D decreased MCP-1 in monocytes incubated with uremic serum. We expected that low levels of MCP-1 would result in fewer leukocytes recruitment and endothelial injury. Cathelicidin is an endogenous antimicrobial peptide^23^ that also works as an inflammatory marker^43^. In the current study, we showed a positive correlation of cathelicidin with IL-6 and TNF-α, reinforcing this hypothesis.

Giffoni *et al*.^33^ demonstrated that lymphocytes obtained from uremic patients supplemented with 25-vitamin D exhibited an increase of VDR and CYP27 expression, resulting in reduced production of pro-inflammatory cytokines. Contrary to expectations, we did not observe an increased intracellular expression of VDR and CYP27 when the monocytes were treated with 25-vitamin D. It supposed that the effect of 25-vitamin D might be dependent on the cell type and exposure time to regulate inflammatory mechanisms.

Our study has some limitations such as the short time of follow-up and the lack of gene expression evaluation, which could help to elucidate the mechanism of anti-inflammatory effect of vitamin D. The strength of our study was to demonstrate the effect of 25-vitamin D in TLR4, cathelicidin and MCP-1 expressions suggesting that this pre-activated form of vitamin D may minimize inflammation. Whether the supplementation of 25-vitamin D in patients with CKD is capable to reduce inflammation needs to be confirmed in further studies.

## Supplementary information

Supplementary Information 
