# Supplementary material for: 25-vitamin D reduces inflammation in uremic environment
Source: Sci Rep. 2020 Jan 10;10:128. doi: 10.1038/s41598-019-56874-1 (PMC6954254; doi:10.1038/s41598-019-56874-1)

**25-vitamin D reduces inflammation in uremic environment**

**Authors:** Rodrigo Barbosa de Oliveira Brito^1^; Jacqueline Ferritto Rebello^1^; Caren Cristina Grabulosa^1^, Walter Pinto^1^; Armando Morales Jr^1^; Rosilene Motta Elias^1,2^; Rosa Maria Affonso Moyses^1,2^; Maria Aparecida Dalboni*^1^

**Institutions:**

1. Universidade Nove de Julho, UNINOVE, Sao Paulo, Brazil; 2. Hospital das Clinicas HCFMUSP, Universidade de Sao Paulo, Sao Paulo, Brazil

**Supplementary Table 1.** ELISA kits

| Protein | KIT |
| --- | --- |
| IL-6 | KIT human IL-6 High Sensitivity cat: HS600B R&D SYSTEMS |
| TNF-α | KIT human TNF-α High Sensitivity cat: HSTA00D R&D SYSTEMS |
| IL-10 | KIT human Il-10 cat: 550613 BD |
| LL37 (Cathelicidin) | KIT human LL37(Cathelicidin) cat: HK321-02 Hycult biotech |
| CCL2 (MCP-1) | KIT human CCL2 (MCP-1) affymetrix eBioscience ref:88-7399-22 |
| NF-kB | Kit human NF-κB p50/p65 Transcription Factor Assay Kit eBioscience |

**Supplementary Table 2.** Description of Monoclonal antibodies used by flow cytometry

| **Tube** | **Monoclonal antibodies** | **Quantity** | **Fluorophore** | **Clone** | **Manufacturer** | **Catalog** |  |
| --- | --- | --- | --- | --- | --- | --- | --- |
| 1 blank | **-** | **-** | **-** | **-** | **-** | **-** |  |
| 2 | CD14 | 5µL | FITC | 61D3 | eBioscience | 11014942 |  |
|  | TLR-4 | 5µL | PE | HTA125 | eBioscience | 12991742 |  |
|  | VDR | 1µL (VDR)  3µL (antibody secondary) | APC | H1512  MOUSE | Santa Cruz/  Invitrogem | SC13133  Z25151 |  |
| 3 | CYP24 | 1µL (CYP24)  3µL (antibody secondary) | Alexa Fluor 488 | D0811  RABBIT | Santa Cruz/  Invitrogem | SC66851  Z5302 |  |
|  | CYP27 | 1µL (CYP27)  3µL (antibody secondary) | Alexa Fluor 647 | K2911  GOAT | Santa Cruz/  Invitrogem | SC49642  Z25608 |  |

**Supplementary Figure 1. Demonstration of expression of CD14, TLR-4 and VDR in U937 cells by flow cytometry.**

Suppl. Fig 1A: U937 cells were selected in the P1 gate according to the characteristics of size (Foward Scatter - FSC) and complexity (Side Scatter - SSC). Suppl. Fig 1B, 1C, and 1D: Autofluorescence adjustment of U937 cells in the detection channels for the respective antibodies labeled with respective fluorochromes FITC, PE and APC. Suppl. Fig1E (P3) = CD14 FITC expression; Suppl. Fig 1F (Q2-2) = Expression of TLR4 PE and CD14 FITC. Suppl. Fig 1G (Q2-6) = Expression of VDR APC and CD14 FITC


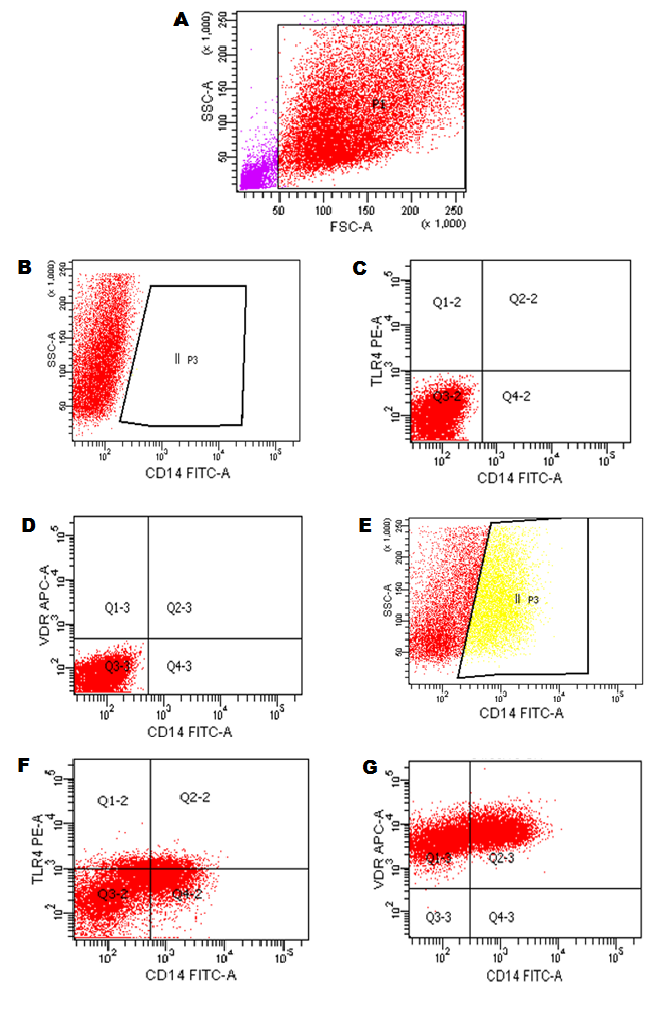
**Supplementary Figure 2. Demonstration of high fluorescence adjustment of U937 cells for analysis of the CYP27 and CYP24.**

Suppl. Fig. 2A: Gate P1 = U937 cells were selected from their Foward Scatter (FSC) and internal complexity (Side Scatter - SSC) characteristics. Histogram-type graphs (number of cells in relation to detected fluorescence intensity) were created from the gate. Suppl. Fig. 2B, 2C: Autofluorescence adjustment of the cells for the detection channels of monoclonal antibodies conjugated to the respective fluorochromes: Alexa fluor 647 (AF 647) and Alexa Fluor 488 (AF 488). Suppl. Fig. 2D, 2E: U937 positive for monoclonal antibodies: CYP27 and CYP24 as shown in the quadrants P3 and P2.


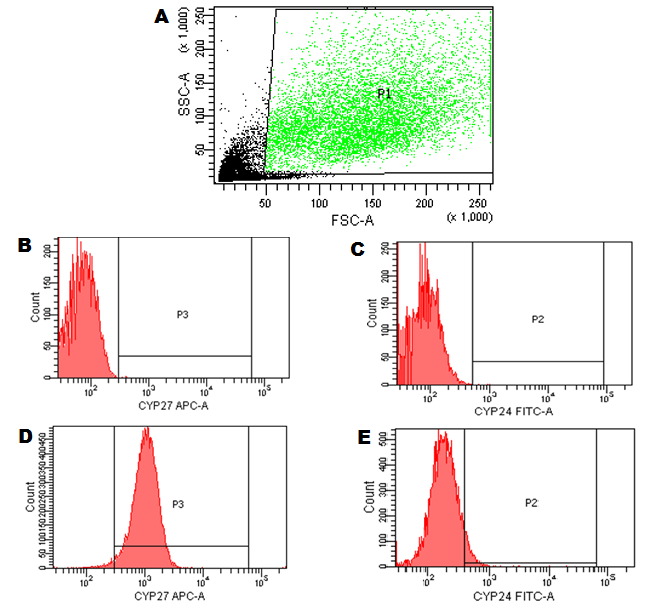


**Supplementary Figure 3. Demonstration of the high fluorescence adjustment of U937 cells for the analysis of Reactive Oxygen Species (ROS).**

Suppl. Fig. 3A: Gate P2 = U937 cells were selected from their Foward Scatter (FSC) and internal complexity (Side Scatter - SSC) characteristics. The histogram type graph (number of cells in relation to the detected fluorescence intensity) was created from the gate. Suppl. Fig. 3B: Autofluorescence adjustment of the cells for the DCFH-(FITC) detection channel. Suppl. Fig. 3C: C: U937 positive for DCFH (FITC) demonstrated in the P2 quadrant.


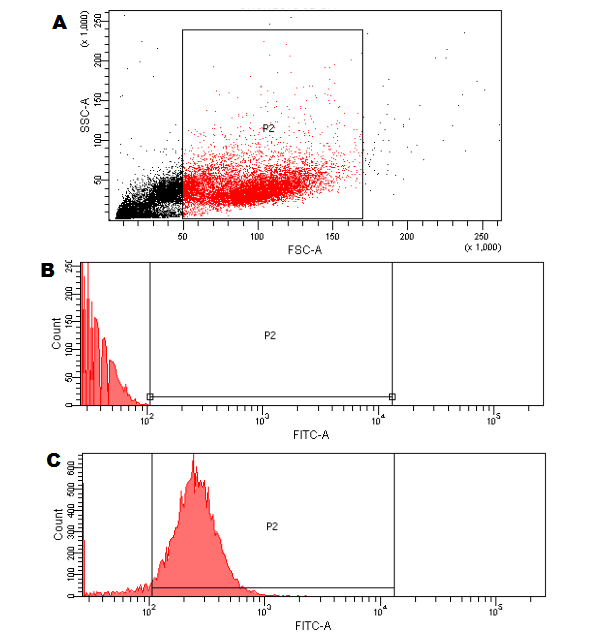

Supplement: Supplementary file 1 — Supplementary Information [file 41598_2019_56874_MOESM1_ESM.docx]
